# Modelling and Optimization of Ultrasound-Assisted Extraction of Phenolic Compounds from Black Quinoa by Response Surface Methodology

**DOI:** 10.3390/molecules26123616

**Published:** 2021-06-12

**Authors:** Valentina Melini, Francesca Melini

**Affiliations:** CREA Research Centre for Food and Nutrition, Via Ardeatina 546, I-00178 Roma, Italy; francesca.melini@crea.gov.it

**Keywords:** quinoa, pseudocereals, phenolic compounds, ultrasound-assisted extraction, response surface methodology, bioactive compounds, phenolic acids, flavonoids, HPLC

## Abstract

Phenolic compounds are currently the most investigated class of functional components in quinoa. However, great variability in their content emerged, because of differences in sample intrinsic and extrinsic characteristics; processing-induced factors; as well as extraction procedures applied. This study aimed to optimize phenolic compound extraction conditions in black quinoa seeds by Response Surface Methodology. An ultrasound-assisted extraction was performed with two different mixtures; and the effect of time; temperature; and sample-to-solvent ratio on total phenolic content (TPC) was investigated. Data were fitted to a second-order polynomial model. Multiple regression analysis and analysis of variance were used to determine the fitness of the model and optimal conditions for TPC. Three-dimensional surface plots were generated from the mathematical models. TPC at optimal conditions was 280.25 ± 3.94 mg of Gallic Acid Equivalent (GAE) 100 g^−1^ dm upon extraction with aqueous methanol/acetone, and 236.37 ± 5.26 mg GAE 100 g^−1^ dm with aqueous ethanol mixture. The phenolic profile of extracts obtained at optimal conditions was also investigated by HPLC. The two extracting procedures did not show different specificities for phenolic compounds but differed in the extraction yield.

## 1. Introduction

Phenolic compounds (PCs) have increasingly become an emerging field of interest in food science and nutrition because of the beneficial effects on human health, associated with their dietary intake. These molecules act as antioxidants by preventing transition metal-mediated formation of hydroxyl free radicals, and by scavenging reactive species of oxygen, nitrogen and chlorine [1]. Long-term consumption of diets rich in plant PCs thus contributes to preventing cardiovascular diseases and some types of cancers [2], as well as protecting against the onset of neurodegenerative diseases [3].

Fruit and vegetables are the main sources of phenolic compounds. However, finding additional products rich in these bioactive compounds has become crucial, in a framework of widening food choices beyond local staples and satisfying consumers’ taste preferences.

Quinoa (*Chenopodium quinoa* Willd.) is singled out as a food rich in antioxidants [4,5], and phenolic compounds showed to be the most investigated class of functional components [4]. PCs have been determined by spectrophotometric assays as total phenolic content (TPC) or by chromatographic techniques that enable one to obtain a phenolic profile. An up-to-date systematic review of the literature showed that spectrophotometric assays have been mainly applied to PC determination in quinoa [4]. Studies on TPC determination in quinoa lack nevertheless optimization of extraction conditions, which is indeed pivotal to obtain accurate data on TPC. The extraction of PCs from the food matrix is a critical step in the determination thereof, since factors, such as the extracting solvent, temperature, time and sample-to-solvent ratio, deeply affect the extraction yield and selectivity. Hence, optimization of extraction conditions is crucial to obtain reliable data.

Response Surface Methodology (RSM) emerged as an effective statistical tool to design experiments, build models, evaluate the effects of factors, and search optimum conditions. It allows overcoming the limitations of single parameter optimization, which is time-consuming and cannot evaluate the complex interactions among the various parameters, as well. RSM has been increasingly used in the optimization of PC extraction from food matrices, especially fruit and vegetables, or by-products thereof [6,7,8,9,10,11]. It allows, in fact, evaluating the effect of independent variables and their interactions on the extraction process. No evidence is available of RSM application to the extraction of PCs in quinoa.

The aim of this study was therefore to model and optimize phenolic compound extraction in black quinoa seeds by RSM. An ultrasound-assisted extraction (UAE) was performed, and the effect of extraction time and temperature, and sample-to-solvent ratio on TPC was investigated. In addition, the effect of solvent nature was studied. In particular, the extraction yield and phenolic profile of methanol/acetone and ethanol aqueous extracts were determined by spectrophotometric and chromatographic methods.

## 2. Results

### 2.1. Optimization of Phenolic Compound Extraction by RSM

RSM was used to identify the optimal conditions for phenolic compound extraction from black quinoa seeds. Extraction time, extraction temperature and sample-to-solvent ratio were set as independent variables, and TPC was set as a response. The study design included testing of two different extracting solutions, namely: (i) methanol/acetone and (ii) ethanol aqueous solutions.

#### 2.1.1. Optimization of Extraction with Methanol/Acetone Aqueous Solutions

TPC in quinoa seed extracts obtained from 15 experiments is listed in Table 1. It ranged between 175.82 ± 5.31 (run 6) and 276.83 ± 6.81 mg GAE 100 g^−1^ dm (run 1). The highest value was observed when extraction was performed for 10 min, at 30 °C and with a sample-to-solvent ratio of 1:20 g mL^−1^. The lowest TPC value was found when quinoa was extracted for 20 min, at 30 °C, and with a sample-to-solvent ratio of 1:5 g mL^−1^.

The data obtained from the Box-Behnken Design (BBD) were fitted to the second-order polynomial equation, and the significance of the model coefficients was determined by analysis of variance (ANOVA). Regression coefficients and corresponding *p* values, indicating the statistical significance of the association between the term and the response, are shown in Table 2.

As regards the model for TPC determined on methanol/acetone aqueous extracts, the linear and quadratic regression coefficients of sample-to-solvent ratio (X_3_) were significant (*p* < 0.05). The linear regression coefficient significantly increased TPC, while the quadratic one had a negative effect. Extraction time (X_1_) and temperature (X_2_), as well as the interaction between all independent variables, had no significant effect on TPC.

The multiple regression analysis of TPC showed that the model was significant (*p* < 0.05). The validity of the model was confirmed by lack of fit testing, as reported in Table 3. ANOVA for the lack of fit test was insignificant (*p* > 0.05), indicating that the model adequately fitted the experimental data. The coefficients of multiple determination (R^2^) revealed a good correlation between the response and the independent variables. The model could explain 96.53% of all variance in data (R^2^_adj_ = 0.90).

Taking into account only the significant factors, the obtained model that shows the relationship between TPC and extraction parameters is described by the following equation:

(1) $$Y=225.28+38.09X_{3}-14.26X_{3}^{2}$$

The statistical significance of the regression equation was checked by Fisher’s F-test. As shown in Table 3, the F-value of regression coefficients was superior to the tabulated value (F_regression_ = 15.47 > F_tabulated(9,5,0.05)_ = 4.77) and the *p*-value was smaller than 0.05, which indicates that the variables of the model have a significant effect on the TPC response at 95% confidence level. In addition, the ratio of the mean square of lack-of-fit and pure error was inferior to the tabulated value (F_lack-of-fit_ = 1.01 < F_tabulated(3,2,0.05)_ = 19.16) which means that the lack of fit statistic was not significant (*p* > 0.05). Hence, the model is valid.

**Figure 1:**
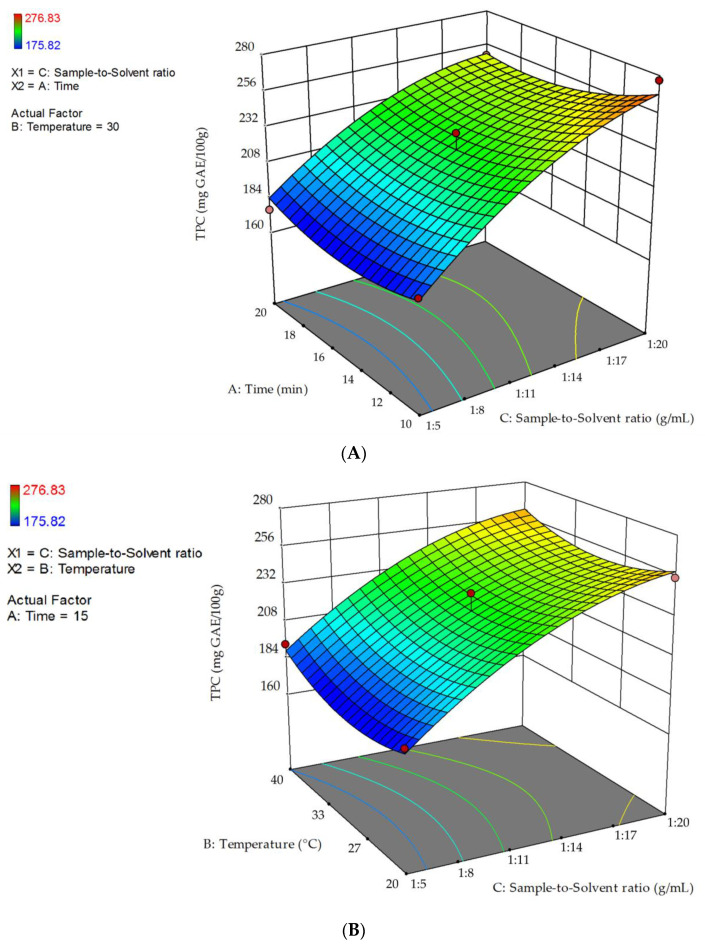
3D response surfaces of TPC as a function of the interaction between the significant factor sample-to-solvent ratio and: (**A**) extraction time, and (**B**) extraction temperature.

Response surface of TPC, as a function of the interaction between the significant variable sample-to-solvent ratio and extraction time or temperature, was plotted (Figure 1). The non-plotted variable is kept at its zero level.

A sample-to-solvent ratio of 1:20 g mL^−1^ allowed obtaining the highest values for the response variable. Figure 1A shows that the highest TPC was obtained at a sample-to-solvent ratio of 1:20 g mL^−1^ and 10 min time. Figure 1B shows that TPC values were higher when a sample-to-solvent ratio of 1:20 g mL^−1^ was used, in relation to higher solid-liquid ratios. TPC decreased from 20 °C up to 29.5 °C and increased at temperature values higher than 29.5 °C.

#### 2.1.2. Optimization of Extraction with Ethanol Aqueous Solutions

TPC in ethanol extracts from black quinoa seeds obtained from 15 experiments is listed in Table 4. TPC ranged between 129.48 ± 2.89 (run 14) and 221.22 ± 2.73 mg GAE 100 g^−1^ dm (run 11). The lowest value was obtained upon extraction for 15 min, at 20 °C and a sample-to-solvent ratio of 1:5 g mL^−1^, while the highest was observed when extraction was performed for 15 min, at 40 °C and a sample-to-solvent ratio of 1:20 g mL^−1^.

Data were fitted to a second-order polynomial equation (Equation (3)), and the significance of the model coefficients was determined by ANOVA. Table 2 shows that linear and quadratic regression coefficients of extraction temperature (X_2_) and sample-to-solvent ratio (X_3_) were significant. The quadratic regression coefficient of the sample-to-solvent ratio had a negative effect on TPC, while the other coefficients had a positive effect. Extraction time (X_1_) and two-way interactions—extraction time * extraction temperature (X_1_X_2_), extraction time * sample-to-solvent ratio (X_1_X_3_) and extraction temperature * sample-to-solvent ratio (X_2_X_3_)—had no significant effects (*p* > 0.05) on TPC of quinoa extracts in ethanol aqueous solutions.

The multiple regression analysis of TPC values showed that the model was significant (*p* < 0.05), and lack of fit testing confirmed the model validity (Table 3). As given in Table 3, ANOVA for the lack of fit test was insignificant (*p* > 0.05). Analysis of variance also pointed out a strong positive correlation (R^2^ = 0.9914) between TPC and significant extraction parameters. Equation (2) shows the mathematical model that describes the relationship between the significant independent variables and the response variable (TPC):

(2) $$Y=160.82+17.11X_{2}+26.00X_{3}+19.78X_{2}{}^{2}-8.50X_{3}{}^{2}$$

The F value of regression coefficients, determined by the Fisher’s F-test, was superior to the tabulated value (F_regression_ = 63.69 > F_tabulated(9,5,0.05)_ = 4.77) and the corresponding *p*-value was smaller than 0.0001. This indicates that the independent variables of the model have a significant effect on the response. In addition, the ratio of the mean square of lack-of-fit and pure error is inferior to the tabulated value (F_lack-of-fit_ = 10.75 < F_tabulated(3,2,0.05)_ = 19.16), and the *p*-value of the lack-of-fit (0.0863) indicates that the model is valid because the lack-of-fit is insignificant.

**Figure 2:**
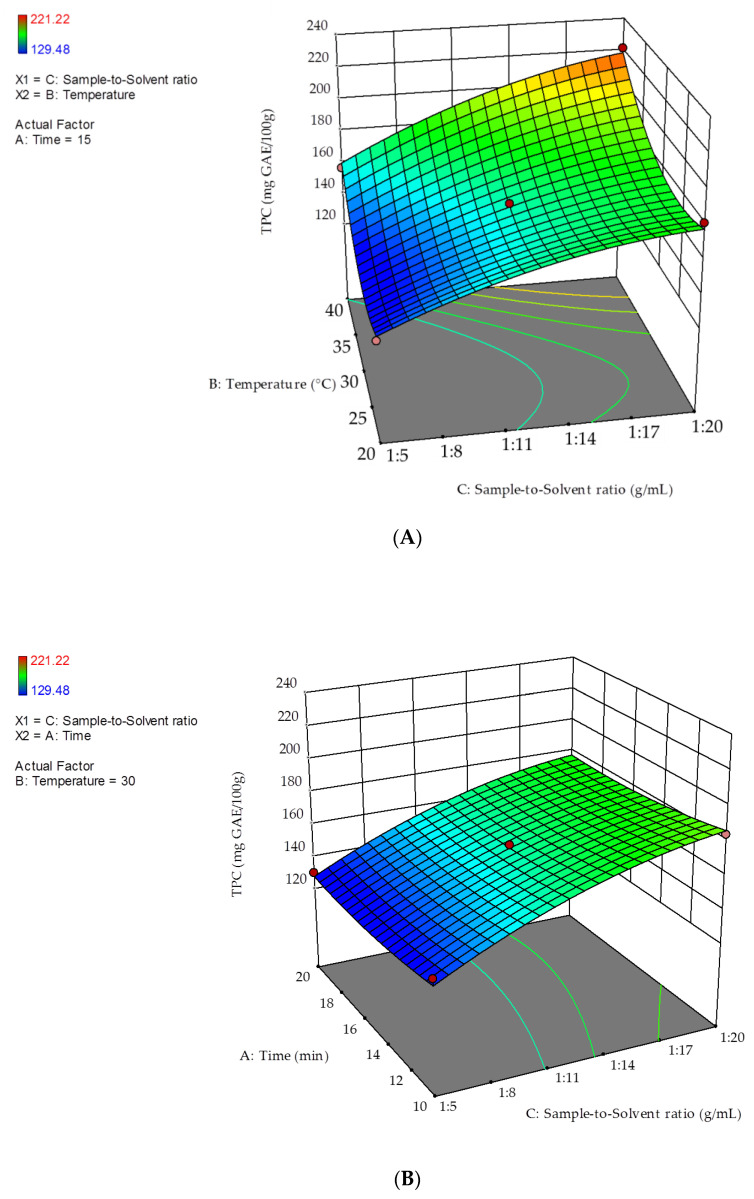

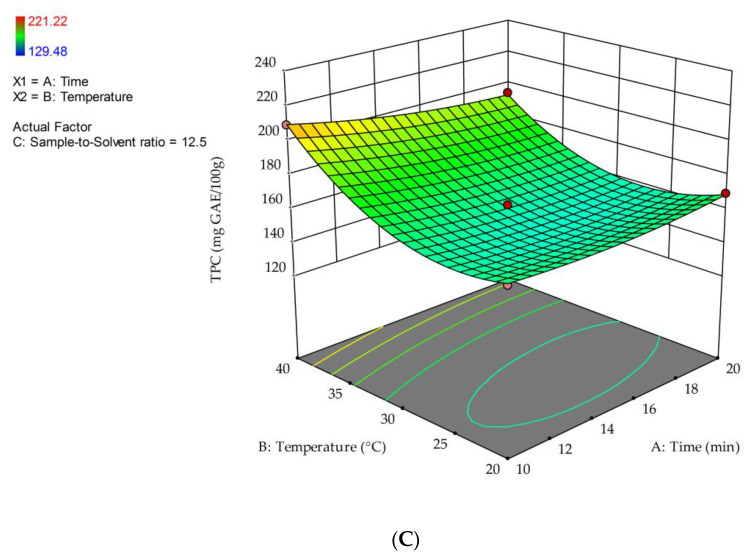
3D response surfaces of TPC as a function of the interactions between extraction temperature and sample-to-solvent ratio (**A**); extraction time and sample-to-solvent ratio (**B**); extraction temperature and extraction time (**C**).

Figure 2 shows the 3D response surfaces of the interactions between the two significant variables (extraction temperature, X_2_, and sample-to-solvent ratio, X_3_) and extraction time. In each panel, the non-plotted variable was kept at its zero level.

Figure 2A shows that the highest TPC value was obtained at 40 °C and a sample-to-solvent ratio of 1:20 g mL^−1^. Figure 2B shows that at the minimum extraction time (10 min), the highest TPC was obtained at a sample-to-solvent ratio of 1:20 g mL^−1^. At any fixed extraction time, the lower the sample-to-solvent ratio was, the higher TPC was. As shown in Figure 2C, at 10 min extraction time, 40 °C temperature should be applied, to obtain the highest response.

#### 2.1.3. Determination and Experimental Validation of The Optimized Conditions

The application of RSM to phenolic compound extraction in black quinoa seeds was targeted to identify the experimental conditions which allow obtaining the highest TPC. A desirability function approach was employed for the maximum yield optimization of the response. Using the RSM-generated model, the optimum experimental conditions to obtain the maximum TPC in methanol/acetone aqueous extracts were 10 min, 20 °C and 1:20 g mL ^−1^. As regards ethanol aqueous extraction, the optimal conditions were 10 min, 40 °C and 1:20 g mL ^−1^. The model for methanol/acetone aqueous extraction estimated a TPC of 282.16 mg GAE 100 g^−1^ dm, and the model for ethanol predicted a TPC value of 231.39 mg GAE 100 g^−1^ dm.

To validate the predicted models, extraction was thus performed at optimal conditions and according to the extraction procedure described for the previous experimental runs.

The mean value obtained for TPC in methanol/acetone extracts was 280.25 ± 3.94 mg GAE 100 g^−1^ dm, which falls within the 95% Confidence Interval (CI; 249.79–316.51 mg GAE 100 g^−1^ dm). The value obtained for TPC in ethanol extracts was 236.37 ± 5.26 mg GAE 100 g^−1^ dm, which falls within the 95% CI (218.81–243.97 mg GAE 100 g^−1^ dm).

The results verify the models and confirm that the settings are the best combination to obtain the highest TPC in quinoa seeds extracted with methanol/acetone or ethanol aqueous solutions.

### 2.2. Phenolic Profile at Optimal Extraction Conditions

**Figure 3:**
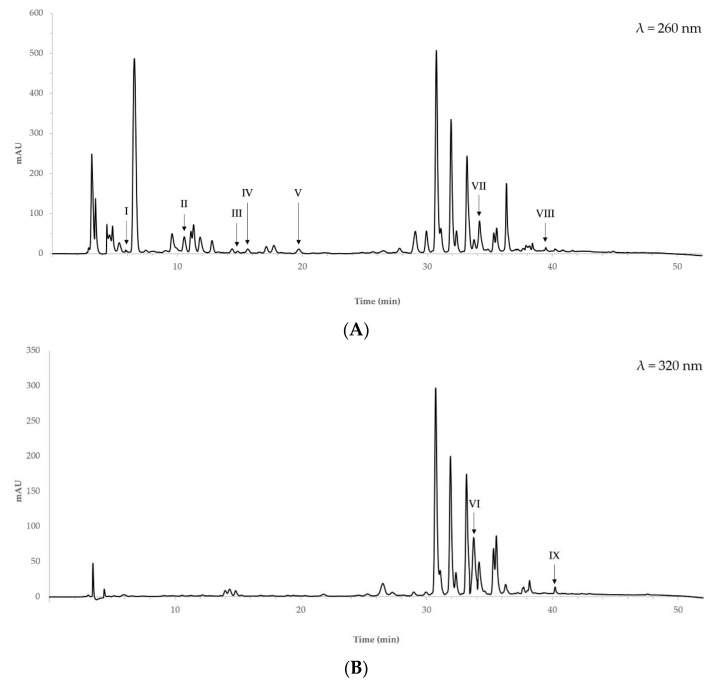
Chromatograms of quinoa phenolic extract in methanol/acetone aqueous solutions at 260 nm (**A**) and 320 nm (**B**). I: gallic acid, II: protocatechuic acid, III: (+)-catechin, IV: 4-hydroxybenzoic acid, V: vanillic acid, VI: *t*-ferulic acid, VII: rutin, VIII: *o*-coumaric acid, IX: 3,4-dimethoxycinnamic acid.

The phenolic profile of quinoa extracts obtained at optimal extraction conditions was determined by HPLC. Figure 3 shows the chromatograms of methanol/acetone extract at 260 and 320 nm.

Gallic acid, protocatechuic acid, (+)-catechin, 4-hydroxybenzoic acid, vanillic acid, *t*-ferulic acid, rutin, *o*-coumaric acid and 3,4-dimethoxycinnamic acid were identified in both methanol/acetone and ethanol aqueous extracts, by comparing retention time and UV spectra of sample peaks with those of pure reference standards. The identified phenolic compounds are listed in Table 5. Based on UV spectra and literature data, two additional peaks, representing 18% and 14% of the total areas, were tentatively assigned as a derivative of quinic acid and quercetin glucosides, respectively.

Caffeic acid, *p*-coumaric acid, sinapic acid, syringic acid, gentisic acid, vanillin and (−)-epicatechin were not detected in the methanol/acetone aqueous extracts nor in the ethanol ones.

The chromatographic profile of the extracts obtained at optimal extraction conditions showed that, as to the identified phenolic compounds, the two procedures did not differ in selectivity for analytes. However, they differed in the recovery yield of some phenolic components. Higher content of protocatechuic acid, (+)-catechin and *t*-ferulic acid was observed at optimal extraction conditions with methanol/acetone aqueous solutions, while a greater concentration of *o*-coumaric and 3,4-dimethoxycinnamic acids was found in ethanol aqueous extracts obtained at optimal extraction conditions.

## 3. Discussion

### 3.1. Total Phenolic Content in Quinoa Seeds

In quinoa, phenolic compounds have been so far quantitated mainly by spectrophotometric methods after reaction with the Folin–Ciocalteu reagent, and results are expressed as TPC [4]. The accuracy and reliability of the quantitation mostly rely on the selection of proper extraction procedures. Solvent nature, sample-to-solvent ratio, temperature and time are some of the factors mostly affecting the extraction process [12]. In this study, RSM was used to model and analyse the effects of these variables on the response (TPC) and to identify the conditions enabling maximum TPC.

As far as the solvent nature is concerned, two different extracting mixtures were tested. A two-step extraction was performed by using aqueous methanol (1st step) and aqueous acetone (2nd step), or by aqueous ethanol (1st and 2nd step). These extracting mixtures have been applied by some Authors to determine phenolic compounds in quinoa or other pigmented grains [13,14,15,16]. Methanol, ethanol and acetone are generally used, because phenolic compounds are more soluble in solvents with intermediate polarities, such as alcohols and acetone, rather than in less polar solvents (e.g., dichloromethane and diethyl ether) [17]. This might be explained by the stereochemistry of phenolic compounds and the intermolecular forces that may occur between the phenolic compound moiety and the solvent. In detail, the electronegative oxygen of ethanol, methanol and acetone can develop hydrogen bonds with the hydroxyl groups of phenolic compounds. Moreover, the aliphatic chain of alcohols may interact with the non-polar fragments inside the phenolic compound molecule [17].

The effect of three different operating conditions, namely, sample-to-solvent ratio, extraction temperature and extraction time, on the response (TPC), was evaluated for each above-mentioned extracting mixture.

As regards the sample-to-solvent ratio, values ranging between 1:4 and 1:40 g mL^−1^ are commonly used in phenolic compound extraction, with the ratio 1:12 g mL^−1^ being the most applied [18]. In this study, three different ratios were tested: 1:5, 1:12.5 and 1:20 g mL^−1^. RSM showed that this variable significantly influenced TPC, whichever extracting mixture was used. It also emerged that the optimal solid-liquid ratio was 1:20 g mL^−1^. This ratio was also used by Pellegrini et al. who determined phenolic compounds in white and pigmented quinoa (75.30–87.60 mg 100 g^−1^ fresh weight) [13], by Stickić et al. who analysed phenolic content in quinoa grown in Europe (56.63–67.86 mg GAE 100 g^−1^ dm) [19], and by some other Authors who determined phenolic compounds that ranged from 40.15 mg GAE 100 g^−1^ dm [20] to 164 mg GAE 100 g^−1^ [21] and 270.99 mg GAE 100 g^−1^ [22] in quinoa samples from South America. Higher ratios (1:10 g mL^−1^) were used by other Authors who found TPC values varying between 15.33 and 43.2 mg GAE 100 g^−1^ dm [15,16,23,24].

The effect of extraction temperature on the response was also investigated. Commonly, in a solid-liquid extraction, high temperatures are applied since they increase the solubility and diffusion of the solute into the solvent. When UAE is performed, low temperatures are generally used. With respect to conventional extracting techniques, the application of ultrasounds improves the penetration of the solvent into the food matrix, thus promoting the solubilization of the solute. Current literature reports extracting temperatures ranging from room temperature to 80 °C in quinoa seeds [13,23,25,26,27]. In this study, low temperatures were tested—20, 30 and 40 °C—since ultrasounds were applied. Room temperature was also used in UAE of phenolic compounds from a white ecotype of quinoa Royal variety grown in India [23], and 15 °C were applied to UAE of quinoa from Morocco [25]. According to RSM analysis, extraction temperatures tested in this study did not significantly influence TPC when aqueous methanol and acetone were used. In contrast, when aqueous ethanol was used, extraction temperature was found to significantly influence the response (TPC). The highest TPC values were observed at 40 °C. However, additional studies could be performed to evaluate if temperatures higher than 40 °C have a positive effect on phenol yield when ultrasounds are applied.

Extraction time may influence the isolation of bioactive compounds, and its setting is strictly linked to the extraction temperature. Commonly, the lower the temperature is, the longer the extraction is [18]. However, compared to conventional techniques, UAE requires a shorter extraction time, since the mass transfer from the matrix into the solvent is accelerated by the cavitation effect. When ultrasonic waves pass through the solvent, cavitation is produced, and vapour bubbles are generated in the liquid (or at liquid–solid interfaces) [28]. The implosion of the vapour bubbles determines an increase in the temperature and pressure of the medium which generate shock waves. They produce an enlargement in the pore walls or the disruption of the cell walls in a short period of time. Hence, there is a reduction of the particle size which allows greater penetration of the solvent into the sample and promotes the release of the target compounds [28]. In this study, three levels of extraction time were tested: 10, 15 and 20 min. The analysis of variance showed that in the models, extraction time did not significantly affect the extraction of phenolic compounds when using aqueous methanol and acetone nor when applying aqueous ethanol. At optimal conditions, extraction was thus performed for 10 min. Based on these results, the application of a wider extraction time range should be explored in further studies, to investigate if longer extraction times can affect significantly the response.

The optimized conditions for phenolic compound extraction with aqueous methanol and acetone enabled to obtain a greater yield than those obtained with aqueous ethanol (280.25 ± 3.94 vs. 236.37 ± 5.26 mg GAE 100 g^−1^ dm). In particular, the same sample-to-solvent ratio (1:20 g mL^−1^) and extraction time (10 min) were applied for both extracting solvent mixtures. In contrast, a higher temperature was required when extracting with aqueous ethanol (40 °C vs. 20 °C). Methanol is mostly used in extracting phytochemicals as it can extract a wide range of compounds with different polarities and has a low boiling point, thus it evaporates in a short time. However, it is worth exploring the use of ethanol because it is safe for operator health and is GRAS. Hence, its use might be explored to recover functional components from agri-food waste to be used as functional ingredients in food preparations [29].

TPC obtained in this study, at the optimal conditions identified by RSM, fell within the range of values reported in the current literature [4]. For black quinoa, TPC varied between 55.5 and 1069.77 mg GAE 100 g^−1^ dm. The lowest content was found in a commercial sample labelled as originating from Peru [30], while the highest was observed in black quinoa grown in the Shanxi province (China) [31]. Intermediate values were also observed [13,27,30,32]. This variability is likely related to (i) sample intrinsic factors such as genotype; (ii) sample extrinsic factors, such as growing conditions, geographical origin, and interaction genotype-by-environment; (iii) processing-induced factors, such as sample storage conditions. Moreover, extraction procedures applied in the above-mentioned studies do not result from an optimization of the response and were mostly retrieved from previous studies. Hence, analytical factors, such as solvent nature, sample-to-solvent ratio, extraction time and temperature could have also contributed to the variability of TPC. As an example, the minimum TPC (55.5 mg GAE 100 g^−1^ dm) was obtained by agitation with ethanol 95% for 1 min followed by soaking for 18h at −4 °C, with a sample-to-solvent ratio of 1:4 g mL^−1^ [30]. The highest value (1069.77 mg GAE 100 g^−1^) was obtained by performing a two-step extraction with aqueous methanol (80%), at 50 °C for 30 min per step, and the sample-to-solvent ratio was 1:20 g mL^−1^ [31]. The application of statistical tools such as RSM is crucial to avoid any under- or over-estimation of TPC. It also allows optimizing resources, that is, achieving the maximum response within a set timeframe and budget, with the minimum usage of the resources themselves. The extraction time and temperature identified in this study for optimal extraction of phenolic compounds are shorter and lower, respectively, than those applied so far to quinoa seeds.

### 3.2. Phenolic Profile

Among the identified phenolic acids, *t*-ferulic acid was the most abundant in both extracts (Table 5). Its content was 4.98 ± 0.08 mg 100 g^−1^ dm at optimal extraction conditions with aqueous methanol/acetone, and 4.11 ± 0.05 mg 100 g^−1^ dm at optimal extraction conditions with aqueous ethanol. Hence, the concentration of *t*-ferulic acid in methanol/acetone aqueous extracts was 21% higher than in the ethanol aqueous extract.

Protocatechuic acid was the second most abundant phenolic acid (Table 5) and the difference in content between the two procedures was approximately 28%, with the highest content in methanol/acetone aqueous extracts. This might be due to a higher solubility of protocatechuic acid in methanol than in ethanol [33]. The occurrence of protocatechuic acid in the aqueous extracts might be also due to the non-enzymatic degradation of anthocyanins, such as cyanidin-3-*O*-glucoside or malvidin glucosides [34]. Ultrasounds can, in fact, induce anthocyanin degradation, since cavitation results in higher temperatures and pressures converting water molecules into free radicals which react with anthocyanins [35].

The occurrence of the above-mentioned phenolic acids was also reported in quinoa samples from China, South America and Europe [19,20,31,36]. Lower values were detected by Liu et al. [31] who found 12.17 µg g^−1^ of *t*-ferulic acid and 9.48 µg g^−1^ of protocatechuic acid in a black quinoa variety from China. In a quinoa sample from Buenos Aires province, 0.57 mg 100 g^−1^ dm of *t*-ferulic acid was determined [16]. Higher values were observed in a quinoa sample of Puno variety grown in Europe (56.21 mg kg^−1^ dm) [19]. In the Chinese quinoa cultivar Jinli-1, higher values of both protocatechuic and ferulic acid were found (126.27 and 118.18 µg g^−1^ dm, respectively) [36]. Differences in protocatechuic and *t*-ferulic content might be due to plant genotypes, growing location and pedoclimatic conditions. In addition, different extraction methods were applied, and this might have greatly affected analytical results, as well. Liu et al. [31] used aqueous methanol (80%) for 30 min at 50 °C; Carciochi et al. [16] performed an ultrasound-assisted extraction with ethanol at 40–60 °C for 60 min; Han et al. [36] used acetone for 5 min at a solvent-to-sample ratio of 25:1; Stikić et al. [19] extracted phenolic compounds with 80% methanol and 50% ethanol under magnetic stirring for 60 min. Compared to the optimal extraction conditions used in this study, longer extraction time and higher temperatures were applied, and that might contribute to phenolic content degradation.

As regards vanillic acid, no significant statistical differences were observed between the two sets of extracts. At optimal extraction conditions with methanol/acetone, vanillic acid content was, in fact, 1.17 ± 0.03 mg 100 g^−1^ dm, and it was 1.12 ± 0.02 mg 100 g^−1^ dm when aqueous ethanol was used (Table 5). Interestingly, the solubility of vanillic acid is higher in methanol than in ethanol, at both 298.15 and 313.15 K and atmospheric pressure [37]. However, the solubility of vanillic acid in ethanol at 313.15 K (40 °C) is comparable to that in methanol at 298.15 K (25 °C). Hence, comparable extraction yield was observed at optimal extraction conditions.

There was no significant statistical difference in the content of gallic and 4-hydroxybenzoic acids between extracts. Both gallic acid and 4-hydroxybenzoic acid are more soluble in ethanol at 313.15 K (40 °C) than in methanol and acetone at 298.15 K (25 °C) [17]. However, the solvent activity coefficient in water is lower at 298.15 K than at 313.15 K. Hence, comparable extraction yields were observed between methanol/acetone and ethanol aqueous extracts.

As regards *o*-coumaric and 3,4-dimethoxycinnamic acids, a greater content at optimal extraction conditions with aqueous ethanol was found. The former showed the highest difference in content between the two procedures. In detail, the use of the aqueous ethanol solution enabled to increase the extraction of *o*-coumaric by 63%. The content in 3,4-dimethoxycinnamic acid in ethanol aqueous extracts was 17% higher than in methanol/acetone extracts.

Among flavonoids, (+)-catechin and rutin were identified and quantified.

(+)-Catechin extraction yield at optimal conditions with aqueous methanol/acetone was higher than with aqueous ethanol (16%). Its content was, in fact, 2.26 ± 0.03 mg 100 g^−1^ dm when aqueous methanol/acetone were used and decreased to 1.95 ± 0.03 mg 100 g^−1^ dm when aqueous ethanol was applied. Methanol has been, in fact, reported as the best solvent for catechin extraction [38].

Among the identified flavonoids, rutin was the most abundant. Its content was not statistically different when ethanol and methanol/acetone aqueous solutions were applied. Lower values are reported in the current literature. In a white quinoa sample from China, the content of rutin was 52.14 µg g^−1^ dm [36]. Compared to data observed in this study, the lower content might be due to differences in quinoa genotype, but also to the use of acetone as extracting solvent. Rutin has, in fact, poorer solubility in acetone than in methanol and ethanol [39].

Overall data show that the two studied extracting mixtures do not have different specificities for phenolic compounds but differ in the extraction yield.

## 4. Materials and Methods

### 4.1. Materials

Dehulled seeds of organic black quinoa (*Chenopodium quinoa* Willd.), labelled as originating from Bolivia (South America) and packed in a protective atmosphere, were purchased in a retailer specialized in organic and biodynamic products in Italy. Proximate composition per 100 g was, as reported in the nutrition label: 380 kcal energy; 6.2 g fat, of which 0.7 g saturates; 65 g carbohydrates, of which 4.0 g sugars; 9.0 g fibre; 12 g protein; and 0.01 g salt.

Folin–Ciocalteu’s Reagent, methanol, ethanol, acetone, acetic acid and acetonitrile were purchased from Carlo Erba Reagents (Milan, Italy).

Caffeic acid, (+)-catechin, *o*-coumaric acid, *p*-coumaric acid, (−)-epicatechin, 3,4-dihydroxycinnamic acid, *t*-ferulic acid, gallic acid, gentisic acid, 4-hydroxybenzoic acid, protocatechuic acid, rutin, sinapic acid, syringic acid, vanillic acid, and vanillin were purchased from Extrasynthèse (Geney, France) and Sigma-Aldrich (St. Louis, MO, USA).

HPLC grade solvents and water purified by a Milli-Q system (Millipore Corp., Billerica, MA, USA) were used in HPLC analysis.

### 4.2. Methods

#### 4.2.1. Sample Preparation

Test quinoa samples were prepared immediately prior to analysis by grinding seeds with a laboratory mill (Janke and Kunkel IKA Labortechnik, Staufen, Germany) provided with a water-cooling system. The obtained powder was sieved with an ASTM woven wire mesh sieve No. 18.

#### 4.2.2. Ultrasound-assisted Extraction of Free Phenolic Compounds

A two-step extraction was performed by coupling ultrasounds and traditional solid-liquid extraction. In detail, a known amount of test quinoa sample (0.25 or 0.4 or 1 g, depending on the experimental design in Table 1) was placed into a PYREX™ screw cap culture tube and added with 5 mL of extracting mixture (methanol:water 80:20 *v*/*v*). The tube was placed in an ultrasound bath system Elmasonic S 100 H (Elma Schmidbauer GmbH, Singen, Germany), operating at 37 kHz, and extraction was performed at different times and temperatures, as reported in Table 1. Ultrasound water bath temperature control was performed, and cold water was added to keep the temperature constant.

After the first extraction step, the solid-liquid solution was refrigerated at +4 °C for 5 min and centrifuged at 7000 rpm for 10 min. The supernatant was collected, then the pellet was added with 5 mL of extracting mixture (acetone:water 70:30 *v*/*v*) and re-extracted at the same conditions applied at the first extraction step. The second-step supernatant was recovered and combined with the corresponding first-step extract. TPC was determined on pooled supernatants.

The above-described ultrasound-assisted extraction of free phenolic compounds was also performed by using ethanol:water 80:20 *v*/*v* as extracting solvent in both extraction steps. Organic solvents/water ratios were selected based on recent studies investigating phenolic compounds in cereals and pseudocereals [13,14,15,16].

An aliquot of the extract was used for TPC determination immediately after the extraction. A known amount of extract was taken to dryness by using a rotary evaporator and stored at −40 °C till HPLC analysis.

#### 4.2.3. Determination of Total Phenolic Content

TPC was determined by colorimetric assay using the Folin–Ciocalteu’s reagent (FCR), as described in Sompong et al. [40]. Briefly, an amount of phenolic extract (120 µL) was placed in a test tube and added with 600 µL of water-diluted FCR (1:10). After three minutes, 960 µL of sodium carbonate (Na_2_CO_3_) 75 g/L was added to adjust pH at 10–10.5. Test tubes were placed at 50 °C for 5 min and the absorbance was measured at 760 nm against the reagent blank. The measurement was compared to a calibration curve of gallic acid in the concentration range of 1.4–14.4 µg mL^−1^. The coefficient of determination (R^2^) of the calibration curve was 0.9982 and the regression equation was y = 0.0871x + 0.0108. Results were expressed as milligrams of Gallic Acid Equivalents per 100 g of sample on a dry matter basis (mg GAE 100 g^−1^ dm).

#### 4.2.4. Experimental Design

The experimental design was established by using the Minitab Pro 18 (Minitab Inc., State College, PA, USA) software DOE package. A three-level-three-factor Box-Behnken Design Response Surface Methodology (BBD-RSM) was used to optimize the conditions of phenolic compound extraction in quinoa seeds.

Extraction time (X_1_; min), extraction temperature (X_2_; °C) and sample-to-solvent ratio (X_3_; g mL^−1^) were set as factors. Three variation levels were considered (Table 6). TPC was set as the response.

A total of fifteen experiments were undertaken (Table 1 and Table 4), with three centre value replications, to establish a model for and optimize the extraction of phenolic compounds with methanol/acetone or ethanol aqueous solutions. All experiments were carried out randomly.

The experimental data were fitted to the following second-order polynomial model equation:

(3) $$Y=\beta_{0}+\overset{3}{\underset{i=1}{\sum }}\beta_{i}X_{i}+\overset{3}{\underset{i=1}{\sum }}\beta_{\mathrm{ii}}X_{i}^{2}+\overset{3}{\underset{i=1}{\sum }}\overset{3}{\underset{j=1}{\sum }}\beta_{\mathrm{ij}}X_{i}X_{j}$$

where Y is the response variable; β_0_ is the regression coefficient for intercept; β_i_, β_ii_ and β_ij_ are the regression coefficients for linear, quadratic and interaction terms, respectively; and X_i_ and X_j_ represent the independent variables. The statistical significance of the terms in the regression equations was evaluated by ANOVA. The terms found as statistically non-significant were excluded from the model. The quality of the fit of the polynomial model equation was expressed by the regression coefficient (R^2^), the F-value of the regression model and the F-value of the lack of fit (LOF) at a probability (*p*) of 0.05. To test the model accuracy, both R^2^ and adjusted R^2^ were estimated. Three-dimensional and contour plots were obtained from regression models.

#### 4.2.5. Validation of the Model

The optimized settings of the independent variables were obtained by maximizing the composite desirability using Minitab Response Optimizer.

These optimal conditions were validated for the maximum TPC, for both extracting solutions. The experimental values were compared with those predicted by the model to assess its validity.

#### 4.2.6. HPLC Analysis of Quinoa Extracts at Optimal Extraction Conditions

The phenolic profile of quinoa extracts obtained at optimal extraction conditions was determined by a Varian ProStar HPLC apparatus (Varian Inc., 2700 Mitchell Drive Walnut Creek, CA 94598, USA) equipped with a binary pump, a photodiode array detector and a column heater. Phenolic compound separation was carried out by an Inertsil^®^ ODS-3 reversed-phase column (250 × 4.6 mm i.d., 5 µm; CPS analitica, Milano, Italy) and elution was obtained by a 52 min gradient. Water acidified with acetic acid (2.5%) (Solvent A) and acetonitrile (Solvent B) were used as mobile phase at a flow rate of 1.0 mL min^−1^. The gradient was set as follows: 3–5% B (0–3 min), 5–10% B (3–8 min), 10% B (8–18 min), 10–20% B (18–30 min), 20–50% B (30–40 min), 50% B (40–45 min), 50–3% B (45–47 min) and hold 3% B (47–52 min). All runs were performed at 40 °C and chromatograms were recorded at 260 and 320 nm.

Phenolic compounds were identified by comparing retention time and UV spectra of sample peaks with those of phenolic compound pure reference standards. Quantification was performed by using calibration curves of pure standards (Table 7).

Galaxie Chromatography Data System software (version 1.9.302.952) was used to control the equipment and process the data. Results were expressed as µg of phenolic acid per g of sample on a dry matter basis.

#### 4.2.7. Statistical Analysis

All statistical analyses were performed with Minitab Pro 18 (Minitab Inc., State College, PA, USA). Microsoft^®^ Excel^®^ for Windows 365 (version 2103) was also used to process experimental data. Design Expert software (version 10, Stat-Ease, Inc., Minneapolis, MN, USA) was used to design contour plots and surface 3D graphs.

## 5. Conclusions

RSM enabled the evaluation of the effect of the extraction time, temperature and sample-to-solvent ratio on phenolic compound content in black quinoa seeds. The optimal setting of extraction conditions was 10 min, 20 °C and 1:20 g mL^−1^ for methanol and acetone aqueous solutions, and 10 min, 40 °C and 1:20 g mL^−1^ for aqueous ethanol. The qualitative composition of the extracts was similar, while statistically significant differences were observed for protocatechuic acid, (+)-catechin, *t*-ferulic, *o*-coumaric and 3,4-dimethoxycinnamic acid content. The study enabled the identification of an analytical procedure for the extraction of phenolic compounds from quinoa seeds based on low volumes of solvents and short time and low energy. It also emphasizes the importance of optimizing the extraction method to avoid inaccurate estimation of phenolic compounds.

## Figures and Tables

**Table 1:** Box-Behnken design applied for phenolic compound extraction from black quinoa seeds with methanol/acetone aqueous solutions: run conditions and measured response (TPC).

Run Order	Independent Variables			Response for Extraction with Methanol/Acetone Aqueous Solutions
	Extraction Time (min), X_1_	Extraction Temperature (°C), X_2_	Sample-to-Solvent Ratio (g mL^−1^), X_3_	TPC (mg GAE 100 g^−1^ dm)
1	10	30	1:20	276.83 ± 6.81
2	15	40	1:5	193.01 ± 5.36
3	10	20	1:12.5	246.03 ± 5.23
4	20	40	1:12.5	253.23 ± 5.95
5	15	30	1:12.5	216.57 ± 7.84
6	20	30	1:5	175.82 ± 5.31
7	20	20	1:12.5	241.93 ± 6.25
8	15	20	1:20	254.93 ± 5.98
9	15	30	1:12.5	223.24 ± 7.01
10	15	40	1:20	258.80 ± 6.26
11	10	40	1:12.5	245.23 ± 6.89
12	20	30	1:20	248.54 ± 5.45
13	15	30	1:12.5	236.03 ± 8.18
14	15	20	1:5	182.32 ± 6.47
15	10	30	1:5	183.25 ± 5.76

**Table 2:** Regression coefficients of the predicted second-order polynomial models for TPC.

	Term	Regression Coefficients	Standard Error	T-Value	*p*-Value
TPC model— methanol/acetone aqueous extraction					
	β_0_	225.28	5.72	39.37	<0.0001
	β_1_	−3.98	3.50	−1.14	0.3078
	β_2_	3.13	3.50	0.89	0.4123
	β_3_	38.09	3.50	10.87	0.0001
	β_1_^2^	10.08	5.16	1.96	0.1079
	β_2_^2^	11.24	5.16	2.18	0.0812
	β_3_^2^	−14.26	5.16	−2.76	0.0397
	β_12_	3.03	4.96	0.61	0.5683
	β_13_	−5.21	4.96	−1.05	0.3408
	β_23_	−1.71	4.96	−0.34	0.7478
TPC model— ethanol aqueous extraction					
	β_0_	160.82	2.39	67.25	<0.0001
	β_1_	−3.21	1.46	−2.19	0.0799
	β_2_	17.11	1.46	11.69	0.0001
	β_3_	26.00	1.46	17.75	<0.0001
	β_1_^2^	3.48	2.16	1.62	0.1670
	β_2_^2^	19.78	2.16	9.18	0.0003
	β_3_^2^	−8.50	2.16	−3.94	0.0109
	β_12_	−5.25	2.07	−2.53	0.0523
	β_13_	−0.99	2.07	−0.48	0.6520
	β_23_	3.25	2.07	1.57	0.1771

**Table 3:** Analysis of variance (ANOVA) of the second-order polynomial models for TPC.

	Source of Variation	DF	Adj SS	Adj MS	F-Value	*p*-Value
TPC model—methanol/acetone aqueous extraction						
	Regression	9	13679.0	1519.9	15.47	0.0038
	Residuals	5	4912	98.2		
	Lack-of-Fit	3	295.6	98.5	1.01	0.5332
	Pure Error	2	195.6	97.8		
	Total	14	14170.2			
	R^2^ = 0.9653					
	adj R^2^ = 0.9029					
						
TPC model—ethanol aqueous extraction						
	Regression	9	9832.36	1092.48	63.69	<0.0001
	Residuals	5	85.77	17.15		
	Lack-of-Fit	3	80.76	26.92	10.75	0.0863
	Pure Error	2	5.01	2.50		
	Total	14	9918.12			
	R^2^ = 0.9914					
	adj R^2^ = 0.9758					

DF: degree of freedom; SS: sum of squares; MS: mean square.

**Table 4:** Box-Behnken design applied for phenolic compound extraction from black quinoa seeds with ethanol aqueous solutions: run conditions and measured response (TPC).

Run Order	Independent Variables			Response for Extraction with Ethanol Aqueous Solutions
	Extraction Time (min), X_1_	Extraction Temperature (°C), X_2_	Sample-to-Solvent Ratio (g mL^−1^), X_3_	TPC (mg GAE 100 g^−1^ dm)
1	10	30	1:20	183.69 ± 2.64
2	15	30	1:12.5	160.45 ± 3.18
3	20	40	1:12.5	193.80 ± 3.10
4	15	20	1:20	181.11 ± 2.88
5	20	20	1:12.5	169.45 ± 2.69
6	20	30	1:20	173.77 ± 2.83
7	15	30	1:12.5	162.55 ± 3.22
8	15	40	1:5	156.58 ± 2.91
9	20	30	1:5	129.90 ± 2.66
10	15	30	1:12.5	159.45 ± 2.58
11	15	40	1:20	221.22 ± 2.73
12	10	40	1:12.5	209.20 ± 2.62
13	10	20	1:12.5	163.86 ± 3.06
14	15	20	1:5	129.48 ± 2.89
15	10	30	1:5	135.85 ± 2.69

**Table 5:** Phenolic compounds identified and quantified in the methanol/acetone and ethanol aqueous extracts. Different letters in the same row represent statistical different results (*p* < 0.05).

Free Phenolic Compound	λ_max_	Rt	Concentration (Methanol/Acetone Aqueous Extract)	Concentration (Ethanol Aqueous Extract)
	*nm*	*min*	*mg 100 g^−1^ dm*	*mg 100 g^−1^ dm*
Gallic acid	270, 228	5.50	0.49 ± 0.01 ^a^	0.52 ± 0.02 ^a^
Protocatechuic acid	291, 257, 228	10.73	3.23 ± 0.05 ^a^	2.52 ± 0.05 ^b^
(+)-Catechin	276, 233	15.21	2.26 ± 0.03 ^a^	1.95 ± 0.03 ^b^
4-Hydroxybenzoic acid	252	15.88	0.65 ± 0.01 ^a^	0.69 ± 0.02 ^a^
Vanillic acid	289, 258	20.12	1.17 ± 0.03 ^a^	1.12 ± 0.02 ^a^
*t*-Ferulic acid	321, 241	34.12	4.98 ± 0.08 ^a^	4.11 ± 0.05 ^b^
Rutin	352, 254	34.45	14.19 ± 0.41 ^a^	15.50 ± 0.34 ^a^
*o*-Coumaric acid	322, 274	38.00	0.24 ± 0.01 ^a^	0.39 ± 0.01 ^b^
3,4-Dimethoxycinnamic acid	318, 245	40.29	0.29 ± 0.01 ^a^	0.34 ± 0.01 ^b^

**Table 6:** Range and levels of experimental variables.

Factors	Symbols		Coded Levels	
		−1	0	+1
Extraction time (min)	X_1_	10	15	20
Extraction temperature (°C)	X_2_	20	30	40
Sample-to-solvent ratio (g mL^−1^)	X_3_	1:5	1:12.5	1:20

**Table 7:** Chromatographic parameters of phenolic compounds analysed by HPLC.

Phenolic Compounds	Regression Equation	R^2^	LOD (µg mL^−1^)	LOQ (µg mL^−1^)
Gallic acid	Y = 0.7961 X + 0.2967	0.992	0.35	1.07
Protocatechuic acid	Y = 1.1062 X + 0.3549	0.991	1.76	5.32
(+)-Catechin	Y = 0.1621 X − 0.0133	0.994	0.99	2.99
4-Hydroxybenzoic acid	Y = 1.4774 X + 0.1550	0.995	0.49	1.48
Vanillic acid	Y = 1.1100 X + 0.2324	0.998	0.38	1.15
*t*-Ferulic acid	Y = 1.6521 X + 0.4750	0.997	1.58	4.79
Rutin	Y = 0.7759 X − 5.3949	0.997	5.21	15.77
*o*-Coumaric acid	Y = 1.3527 X + 0.2021	0.999	0.16	0.49
3,4-Dimethoxycinnamic acid	Y = 1.4616 X + 0.1248	0.996	0.24	0.73

LOD: Limit of Detection; LOQ: Limit of quantification.

## Data Availability

The data presented in this study are available on request from the corresponding author.

## Sample Availability

Not available.
